# The Relative Value of Turtle Tuberculin in the Treatment of Tuberculosis*Author’s abstract of an article under this head, in the *N. Y. Medical Journal*, September 11, 1913, by Dr. Edward E. Myers, 418 Central Park, West, New York.

**Published:** 1913-10

**Authors:** 


### TEXAS MEDICAL JOURNAL.

Established July, 1885

F. E. DANIEL, M. D., - - - - Editor, Publisher and Proprietor

Published Monthly.—Subscription, $1.00 a Year.

VOL. XXIX. AUSTIN, OCTOBER, 1913. No. 4

The publisher is not responsible for the views of the contributors.

### Original Articles.

For Texas Medical Journal.

### The Relative Value of Turtle Tuberculin in the Treatment of Tuberculosis.*

"The treatment of individual diseases with medicines or by methods having a selective curative action has until recent years been limited. With the establishment of the germ theory, and vaccine therapy of certain diseases and the development of information concerning immunity, new methods of specific treatment have been made possible, and are now practiced under the terms of serum and vaccine therapy." This is part of an introductory paragraph of a valuable contribution on the above subject appearing in the *New York Medical Journal*, for September 13, 1913, by Doctors J. W. Beattie of New Hampshire and E. E. Meyers, of 418 Central Park West, New York City.

The authors mention the fact that to Robert Koch belongs the honor of giving to the world twenty-three years ago tuberculin, which was the first great advance in the diagnosis of tuberculosis. Prior to this, the disease was generally recognized as a fatal malady; it was not diagnosed until the disease was advanced and the symptoms marked and then death was required to substantiate the diagnosis. His discovery of the difference in the action of the remedy on the healthy and the tuberculous has proven to be one of the most important discoveries in the modern study of tuberculosis. This discovery gave the profession the tuberculin test which has

*Author's abstract of an article under this head, in the *N. Y. Medical Journal*, September 11, 1913, by Dr. Edward E. Myers, 418 Central Park, West, New York.

not only made possible an early diagnosis of the presence of tuberculosis, but has also given us a more thorough understanding of the nature of the disease and the essentials of its prevention, as well as led to its specific treatment.

Drs. Beattie and Myers quote von Ruck's reference to the claims of Friedman for the superior value of a living tubercle bacilli in the treatment of tuberculosis, and deprecates the Berlin doctor's spectacular advertising propaganda in the daily press. Von Ruck said, "Inasmuch as living tubercle bacilli of the human type have been found in vaccinated cattle both in their flesh and in their milk, as long as three years after their intravenous injection, the objection to the use of the living tubercle bacilli as an antigen, or vaccine for prophylactic purposes in the human subject is well founded. A more formidable objection is, however, the danger of virulence."

They aver Prof. Piorkowski, working along the lines of Prof. Koch's discovery, isolated a living antigen in the form of tubercle bacilli recovered from a turtle, as far back as 1903 without in any manner questioning its non-virulence. Since that time he has continued his research along this line, and has at last succeeded in perfecting a tuberculin produced from the tubercle bacilli of a deep sea turtle which is non-virulent, and with which, he has successfully experimented with thousands of cases during the past few years at his laboratory in Berlin.

Further quoting Piorkowski, the authors refer to his lecture delivered at the Royal Hospital for the Diseases of the Chest, London, Eng., on April 1, 1913. (1 *British Journal of Tuberculin*, July issue, 1913.) On discussing his turtle tuberculin, Piorkowski said, "We must differentiate between mammals which produce their offspring alive, and the class to which human beings and oxen belong; and birds—that is, animals which lay eggs, thirdly, reptiles, which possess horny or long integument and also lay eggs. Lizards, crocodiles and turtles belong to that last class. Finally, we have to think of fishes which breathe as long as they are young through gills or by their lungs, and also lay eggs. We thus see very clearly that resemblances are to be found only among lung-breathing animals, and it is for this reason, probably, that the results described are obtained on the injection of tubercle bacilli of similar kind. It became very evident that turtles were especially adapted for our purpose."

In further describing his work along this line Piorkowski says, "It is very noteworthy that the turtle tubercle baccillus in its further behavior, both culturally and morphologically, displayed an extraordinary resemblance to the human tubercle bacillus. Its growth at 37 degrees F. is remarkably characteristic. The main point about this strain is that it can be used without risk of any manifestations—a circumstance which may be ascribed to the fact that for the last ten years it has been reinoculated afresh daily, and thus has acquired generally an extraordinary innocuousness, becoming both avirulent and atoxic."

The authors in explaining the biological action of Piorkowski's turtle tuberculin quote the latter as follows: "Let us for example, consider atoxic action a little more closely. When a poison enters the body—e. g., tubercle toxin—the first point concerns the existence of receptors which can take up the tubercular poison. If these do not exist, no infection by tubercle bacilli can occur, for the organism possesses congenital immunity towards the action of these bacilli.

The harmless turtle tuberculous toxin combines with the receptors, and the combination is thrown off into the blood as antoxin. New receptors are formed in large quantity, but they are capable of seizing not only the turtle tubercle bacilli, with which they have been hitherto dealing, but also human bacilli, and thus render them harmless. If there is a profuse formation of new receptors, and if the human tubercle bacilli have increased unduly, complete recovery may be affected. The rationale of the cure is along these lines. There is also the additional advantage that turtle tubercle bacilli are innocuous and harmless, and therefore this method is especially well adapted for protecting inoculation.

Recent investigations with turtle tuberculin, in Prof. Piorkowski's laboratories, made by the authors show that tubercle bacilli, when grown in the blood serum of (cold blooded animals) turtles change quite distinctively its bacteriological characteristics, particularly in lessening its virulence and at the same time increasing its power to form antibodies in the blood of tuberculous patients. This turtle tuberculin acts as a direct stimulant to the antibodies of tuberculosis, exerting far greater beneficial effects than human tuberculin, even when the latter is given in the most carefully graded and guarded doses. Furthermore, turtle tuberculin produces only a very slight reaction, besides it possesses far greater immunizing properties than does human tuberculin with none of the latter's untoward effects.

According to the authors' experience, the smallest immunizing dose was one minim of turtle tuberculin administered in 16 minims of normal salt solution. The interval between doses depends upon the recurrence or exacerbation of original symptoms, which is usually about seven days. Very slight reactions such as a rise of temperature to 100° F., and more or less langour for about twenty-four hours following the injection are the only reactions which occur even with a maximum dose.

The best site for injection of turtle tuberculin is in the fold of the gluteal region between the glutens maximus and minimus muscles which location facilitates absorption.

In closing the authors make the following comparisons:

LOCAL REACTION

*Human Tuberculin.*—Redness and infiltration begin in area of injection in from four to eight hours. No thickening of the skin. Area of infiltration usually very tender. Abscess sometimes follows injection. Adjacent lymph glands swollen. Doses smaller. Effect slower. Reaction marked. Length of treatment prolonged.

*Hygienic Treatment.*—Not always feasible. Treatment prolonged. Necessitating interference with daily avocation. Results not always satisfactory. Recurrence frequent.

*Turtle Tuberculin.*—Redness and infiltration begin in area of injection in twelve hours. Slight elevation and thickening of skin. Area of infiltration is not tender. No abscess follows at point where the needle pierces skin. Lymph glands not swollen. Dosage greater. Effect more rapid. Reaction slight. Length of treatment short.

*Turtle Tuberculin Treatment.*—Always feasible. Treatment shortened. Does not interfere with daily avocation. Results very encouraging. Recurrence improbable.

---

In lumbar kidney operations take pains to protect the ilio-hypogastric nerve. Its division causes paralysis of a considerable area of the abdominal wall and produces a distressing pseudo-hernia. If the nerve is divided in the operation suture it.—*American Journal of Surgery.*

---

Philanthropist.—Er—I sent a poor starving devil down to you with a note this morning to tell you to give him a meal. What's the bill?"

Innkeeper—Eighteen pence.

Philanthropist—What are the items?

Innkeeper—Four beers and two cigars.—*Sydney Bulletin.*

